# The Effect of Maltose on Structural, Physicochemical, and Digestive Properties of Lentil Starch under Electron Beam Irradiation

**DOI:** 10.3390/foods13162544

**Published:** 2024-08-15

**Authors:** Danyang Liang, Qing Liu, Haiyu Luo, Lin Luo, Khamiddolov Temirlan, Wenhao Li

**Affiliations:** College of Food Science and Engineering, Northwest A&F University, 22 Xinong Road, Yangling District, Xianyang 712100, China; liangmmsheep@163.com (D.L.); liuqing2218699479@163.com (Q.L.); lhy107823@163.com (H.L.); lluo6@unl.edu (L.L.); khamiddolov.t@mail.ru (K.T.)

**Keywords:** lentil starch, electron beam irradiation, maltose, multiscale structure, in vitro digestibility

## Abstract

This study investigated the effects of electron beam irradiation (EBI) on the structural, physicochemical, and functional properties of lentil starch with varying maltose content. EBI did not significantly disrupt the starch’s surface structure or cause amorphization of starch and maltose crystals, but it significantly reduced the intensity of starch’s XRD peaks. The presence of maltose intensified internal growth ring damage, leading to more cross-link and rearrangement between short chains, improving short-range ordering of lentil starch and enhancing starch’s solubility and thermal stability. Additionally, adding maltose that EBI then treats can lead to an increased content of slowly digestible starch in samples.

## 1. Introduction

Due to its wide availability, diverse applications, and biodegradable and renewable characteristics, starch is extensively used in the food, chemical, pharmaceutical, and biological fields [1]. Lentil (*Lens culinaris Medic*) is a legume with high starch content, making up about 46.3% of its composition [2], which is also rich in potassium, essential amino acids, insoluble dietary fiber, and polyphenols [3], making them a valuable gluten-free ingredient in the food processing industry. Despite its potential, lentil starch is not widely used in industrial production, especially in the food industry, because of the inherent properties of native starches, such as poor solubility and intolerance to mechanical shear [4]. Therefore, modifying the properties of lentil starch can enhance its physical, chemical, and functional characteristics, making it more suitable for industrial applications. Starch modification methods are categorized into physical, chemical, and biological processes. Chemical modification often results in chemical residues and environmental issues due to wastewater generation. Although enzyme modification, a biological modification, can efficiently produce modified starch, it is limited in practical applications due to its time-consuming and complex processes [5].

Electron beam irradiation (EBI) is a novel physical modification technology that has gained popularity in production due to its controllability and nonthermal processability. EBI is valued for its rapidity, high efficiency, environmental friendliness, absence of chemical residues, and low heat generation [6,7,8,9]. EBI’s etching effect on the starch surface within permissible food processing doses is weak, generally not causing significant damage to starch granules [10]. However, EBI significantly impacts the internal structure of starch. It can cleave α-1,6-glycosidic bonds, forming more linear short chains [10]. Given starch granules’ “onion-like” semi-crystalline structure [11], breaking glycosidic bonds leads to structural changes in both amorphous and crystalline regions. As a result, EBI-treated starches typically show a decrease in relative crystallinity, enthalpy of melting [10], molecular weight [12], and the proportion of long chains [9]. These structural degradations further induce changes in physicochemical and functional properties, such as reduced starch viscosity [13] and increased amylase sensitivity [14]. The EBI irradiation dose and frequency significantly affect starch structure [9]. Additionally, the material’s composition, such as moisture content [15] and NaCl content [16], also influences the production and properties of irradiation-modified starch. In starch-based foods, EBI treatment of the main components and starch can change food quality and nutritional properties. Using natural ingredients commonly found in food as a medium for EBI ensures that the modified starch is safer and more suitable for the food industry.

In industrial food production, it is challenging to treat specific substances individually due to the complex composition of food systems. Maltose, a common sweetening ingredient, is prevalent in starch-based foods in various forms. When EBI treatment is used as a biocidal technique in food processing, the amount of maltose may affect the effectiveness of EBI, particularly on starch, the main component. This study aimed to investigate the effects of maltose on the structural, physicochemical, and functional properties of lentil starch at different EBI doses. By adding maltose in varying proportions to lentil starch and subjecting it to different EBI doses, this study explored maltose’s potential as a natural ingredient in the processing of EBI-modified starch. It is speculated that maltose may influence EBI-modified starch, causing different degrees of color change, promoting more cross-linking between starch molecules, and slowing the decrease in thermal stability due to EBI. Additionally, maltose may also improve starch’s hydration capacity during gel formation. This study expands the potential applications of lentils and their irradiation-modified starch products. It elucidates the interactions between maltose and starch during EBI and guides EBI technology’s promotion in starch-based food processing.

## 2. Materials and Methods

### 2.1. Material

Lentils (*Lens culinaris Medic*) were procured from a local market in Yangling, China. All chemical reagents used in this study were of analytical grade and sourced from Aladdin Biochemical Technology Co., Shanghai, China.

### 2.2. Starch Isolation

The lentils were soaked in distilled water for 12 h and then pulverized using a pulverizer (L13-Y91, Jiuyang Liability Co., Ltd., Guangzhou, China), following the method described by Ge et al. [17]. The resulting slurry was filtered through a 100-mesh nylon sieve, and the extract was left to settle for 2 h before the upper transparent liquid layer was poured off. The residue was rewashed with distilled water, repeating the process three times. The extract was then centrifuged at 3500 rpm for 15 min. The supernatant and emulsion layer were discarded. The purified starch isolate was dried at 45 °C for 24 h. The dried starch was then pulverized and passed through a 100-mesh sieve, resulting in raw lentil starch with a dry basis moisture content of 11.9%, protein content of 0.17%, and fat content of 0.09%.

### 2.3. EBI Treatment

Dividing lentil starch into nine portions of 100 g each, 5% and 10% analytically pure maltose was added to six portions. To thoroughly mix the maltose and lentil starch, 300 mL of distilled water was added to each portion to create a starch emulsion. Then, the samples were dried in a hot air oven at 45 °C, then ground and sieved using a 100-mesh nylon sieve.

Starch samples were irradiated at Hesheng Electron Irradiation Co. Ltd. (Yangling, China). Each sample (50 g) was packed in PE bags (8 cm × 12 cm) and irradiated using a 15 MeV/150 kW high-energy electron linear accelerator at absorbed 4.0 and 8.0 kGy. The samples were controlled: E4; E8; M5; M5E4; M5E8; M10; M10E4; and M10E8.

### 2.4. Characterization of Microstructure

#### 2.4.1. Scanning Electron Microscopy (SEM)

Following the method described by Lei et al. [18] with slight modifications, the starch samples were fixed onto aluminum plates using double-sided carbon tape and then coated with a gold film under vacuum. The microscopic morphology of the lentil starch granules was examined using a scanning electron microscope (S-3400 N, Hitachi High-Technologies Corporation, Tokyo, Japan) under an accelerating voltage of 15.0 kV and at a magnification of 2000×.

#### 2.4.2. Confocal Laser Scanning Microscopy (CLSM)

A total of 2 mg of starch was mixed with 4 μL of 8.0 mM APTS solution (using acetic acid as the solvent) and 4 μL of 1.0 M sodium cyanoborohydride, stained for 24 h at 30 °C. After the stained samples were washed, they were put on a glass slide with a glycerol–water mixture (1:1, *v*/*v*). A small amount of the stained sample was dispersed in the glycerol–water mixture and placed on a glass slide to observe the growth rings. The samples were photographed using a confocal laser scanning microscope (CLSM) with a 100× oil immersion lens (LEICA TCS SP8, Leica Microsystems GmbH, Wetzlar, Germany). The dye excitation wavelength was set to 488 nm.

#### 2.4.3. Polarized Light Microscopy Structures

Analyzing starch’s polarizing light by polarizing light microscope (B × 53, Motic Olympus Co., Ltd., Xiamen, China), it was applied dropwise to a glass slide by combining the starch sample with a glycerol–water solution (1:1, *v*/*v*). The slides were then examined under the microscope to observe the characteristic Maltese cross pattern of starch under polarized light at 400× magnification.

### 2.5. Determination of the Crystalline Structure

#### 2.5.1. X-ray Diffraction Analysis (XRD)

An X-ray diffractometer (Rigaku D/max2200pc, Rigaku Co., Ltd., Tokyo, Japan) was used to analyze starch. A small amount of starch was weighed and added to the instrument sample tray. The test parameters were set as follows: a 40 kV accelerating voltage and 30 mA current were used, the diffraction pattern 2θ range was set at 4° to 60°, the scanning speed was set to 4°/min, and the step size was 0.02°.

#### 2.5.2. FT-IR Analysis

The starch samples were measured using a Fourier Transform Infrared Spectrometer (Vertex 70, Bruker Co., Ltd., Berlin, Germany), weighing 1.0 mg sample and mixing with 100 mg of KBr. After grinding and pressing the mixture, it was placed in the FT-IR instrument with the following parameters: TR mode, pure KBr as the blank control, and the samples were scanned at 400 to 4000 cm^−1^ with a spectral resolution of 4 cm^−1^.

### 2.6. Chromaticity Value

The chroma of samples were measured using a spectrophotometer (CS600, Hangzhou CHNSpec Technology Co., Ltd., Hangzhou, China) separately; a quantity of starch was weighed and placed flat in a plastic bag. After calibrating the colorimeter with a black-and-white plate, a 10 mm reflective ring was selected and the chromaticity value measured at a randomly selected location. The color difference (ΔE) from the co-ordinates of the color was calculated by applying the following equation [19]:

(1) $${\Delta E}^{*}=\sqrt{{\left({∆L}^{*}\right)}^{2}+{\left({∆a}^{*}\right)}^{2}+{\left({∆b}^{*}\right)}^{2}}$$

In the formula, ∆L*, ∆a*, and ∆b* denote the color difference between the sample and the control sample, respectively.

### 2.7. Pasting Properties

A rapid viscous analyzer measured starch’s pasting properties (RVA-Master, Newport Scientific Co., Ltd., Warriewood, Australia). According to the result calculated by the software, 2.92 g starch (dry basis) was weighed and then dispersed in an aluminum box with 25 mL of distilled water. It was then placed in the RVA device for determination. Test procedure parameters were as follows: heating the sample to 50 °C for 1 min, raising the temperature to 95 °C over 3.75 min, and maintaining it at 95 °C for 2 min. Then, it was reduced to 50 °C over 3.75 min and maintained at 50 °C for 2 min. The following parameters were recorded: peak viscosity (PV), trough viscosity (TV), breakdown (BD), final viscosity (FV), setback (SB), and pasting temperature (PT).

### 2.8. Thermal Properties

A Differential Scanning Calorimeter (Q2000, TA Instruments Co., Ltd., New Castle, DE, USA) was used to determine the thermal properties of the samples. In detail, 3.0 mg of starch was weighed and mixed with 9 μL distilled water in an aluminum crucible. After 12 h of equilibration, the crucible was placed in the DSC apparatus and an empty crucible was used as a control. The parameters were equilibrated at 20 °C, isothermal for 2 min, heated to 120 °C at 10 °C/min, and a sample purge flow rate (N_2_) of 50.0 mL/min.

### 2.9. Solubility and Swelling Power

The solubility and swelling power of the samples were determined from 50 °C to 90 °C following the method of Liang et al. [9]. Precisely weighed 0.5 g of starch was placed in a 50 mL centrifuge tube, and 25 mL of distilled water was added to create a 2% starch suspension. After thorough mixing, the suspension was shaken at 50 °C, 60 °C, 70 °C, 80 °C, and 90 °C for 30 min, cooled to room temperature, and centrifuged at 3500 rpm for 10 min. The supernatant was collected in an aluminum box, dried at 105 °C in a hot air oven to a constant weight, and weighed as W1. The precipitate in the test tube was weighed as W2. The calculation formulas were as follows:

(2) Solubility (S,%)=W1/W×100

(3) $$Swelling\;power\;\left(SP,g/g\right)=\left[W2/\left(W-W1\right)\right]$$

W is the weight of samples (g); W1 is the weight of the supernatant in the aluminum box (g); and W2 is the weight of the residue after drying the aluminum box (g).

### 2.10. In Vitro Digestibility

The in vitro digestibility of starch was tested concerning the method Englyst et al. [20] described, with minor modifications. A 100 mg starch sample was weighed and added to a 50 mL centrifuge tube, followed by 10 mL of 0.5 M acetate–sodium acetate buffer at pH 5.2. The mixture was incubated at 37 °C for 10 min and then 4 mL of 3000 U/mL porcine pancreatic α-amylase and 1 mL of 2500 U/mL amyloglucosidase were added sequentially. The mixtures were then allowed to react for 20 min and 120 min, respectively. Immediately after the reaction, the enzymes were inactivated by boiling the samples in water for 5 min. The supernatant was collected by centrifugation at 3500 rpm for 10 min. The DNS (dinitrosalicylic acid) colorimetric method determined the released glucose content. The contents of rapidly digestible starch (RDS), slowly digestible starch (SDS), and resistant starch (RS) were calculated according to the following formula:

(4) RDS (%)=[(G20−F)×0.9]/T 

(5) SDS (%)=[(G120−G20)×0.9]/T

(6) $$RS\;\left(\%\right)=1-RDS\left(\%\right)-SDS\left(\%\right)$$

G20 and G120 are the glucose content after undergoing 20 min and 120 min hydrolysis; F is the free glucose content; and T is the total starch content.

### 2.11. Statistical Analysis

The data are presented as mean ± SD with three replicates per group. Statistical analysis was conducted using SPSS 21.0 (SPSS Inc., Chicago, IL, USA). A significance level of *p* < 0.05 was employed for ANOVA and Duncan’s test. The data underwent principal component analysis (PCA) and Pearson’s test using Hiplot (Shanghai Teng Yun Cloud Plotting System, Shanghai, China).

## 3. Results

### 3.1. Morphological Structure

#### 3.1.1. SEM

**Figure 1:**
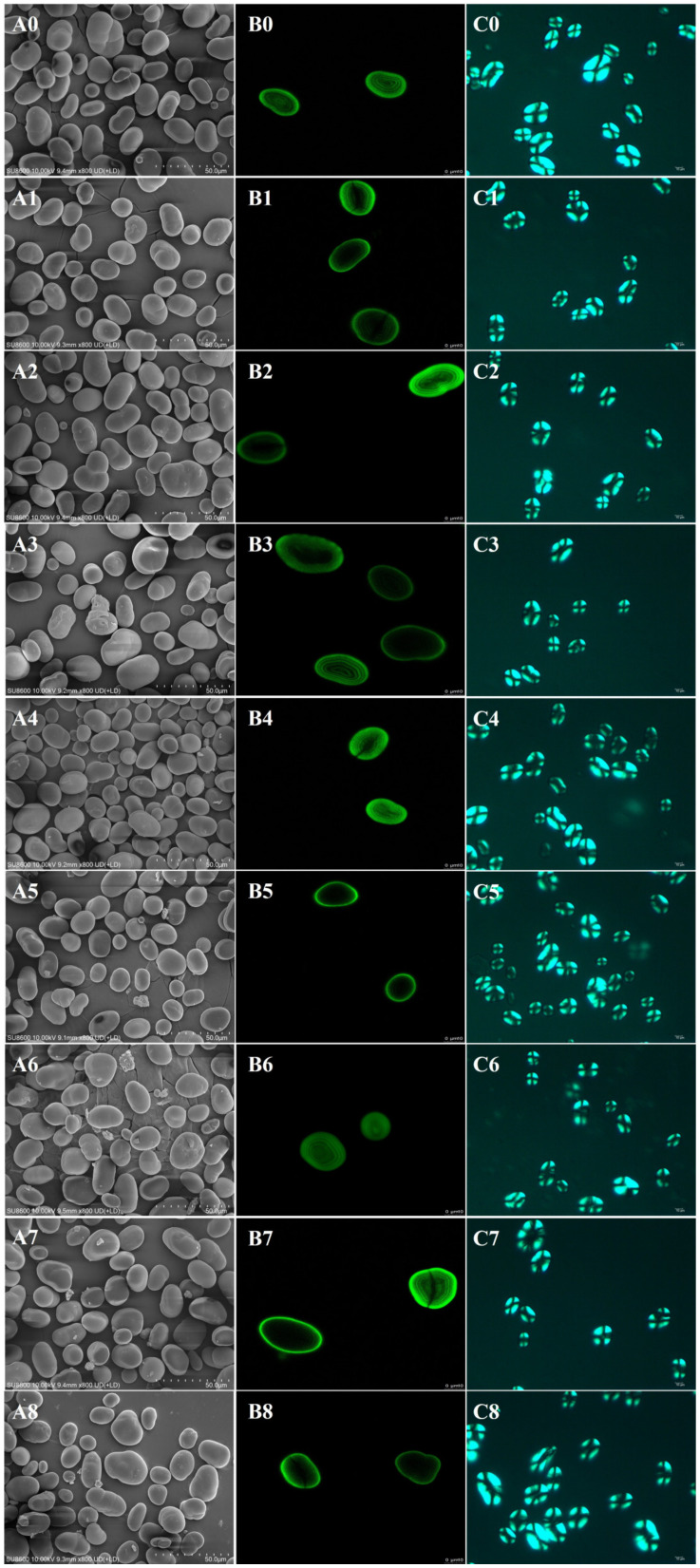
Scanning electron micrographs (SEM) (×800) (**A**), confocal laser scanning micrographs (CLSM) (1024 × 1024-pixel resolution) (**B**), and polarized light micrographs (**C**) (×400) of lentil starch treated with electron beam irradiation at different maltose addition proportions. For 0, Native; 1, E4; 2, E8; 3, M5; 4, M5E4; 5, M5E8; 6, M10; 7, M10E4; 8, M10E8, respectively.

The surface morphology of starch granules observed by SEM is shown in Figure 1(A0–A8). Consistent with the findings of Li et al. [21], the native lentil starch granules were elliptical and kidney-shaped with smooth surfaces. After EBI treatment, especially at higher irradiation doses (8 kGy), it showed aggregation and cross-linking between some amount of the granules, in addition to an increase in surface roughness. The surface roughness of starch-containing maltose also changed after EBI treatment at different doses. The degree of change in the surface morphology of lentil starch with 10% maltose added was more pronounced at an EBI treatment dose of 8 kGy. In addition to the effect of EBI, this may be related to the partial melting of maltose due to the thermal effect produced by irradiation or the degradation of maltose due to the action of free radicals [22].

#### 3.1.2. CLSM

CLSM images of native lentil starch and irradiation-treated starch are shown in Figure 1(B0–B8). The intact “onion-like” starch growth rings were observed in the native starch without EBI treatment and in the samples M5 and M10. Changes in fluorescence intensity in the CLSM images reflect the changes in the number of starch-reducing ends after irradiation treatment [23]. With the increase in the EBI treatment dose, all samples’ starch growth ring structures were damaged to different degrees, mainly manifested by the fracture of the growth ring structure, the disappearance of the internal structure, and the enhancement of the fluorescence intensity in the photographs. These results were the same as previous studies on wheat and potato starch [9,16]. At the same irradiation dose, the growth ring annulus structure was more blurred when the maltose addition was 10%.

#### 3.1.3. Polarized Light

Images of starch granules are shown in Figure 1(C0–C8) under polarized light microscopy. In native lentil starch, a distinct and structurally complete Maltese cross pattern is observed. The addition of maltose alone does not affect the brightness or morphology of these polarized crosses. However, significant changes occur following EBI treatment. After irradiation, although the polarized crosses remain visible, the hilum structure at the center of some starch granules is noticeably disrupted, accompanied by partial darkening and more pronounced changes in the shape of the Maltese crosses. These effects are more evident in samples treated with EBI after adding maltose.

Similar changes in the darkness and shape of Maltese crosses have been observed in enzymatically digested sweet potato starch [17], attributed to the enzyme’s destructive effect on the starch crystal structure. It is known that free radicals generated by EBI can cause the disintegration of starch crystals, increasing their overall disorder [18]. The presence of maltose appears further to amplify the degrading effect of EBI on starch.

### 3.2. Crystalline Structure

#### 3.2.1. FT−IR

**Figure 2:**
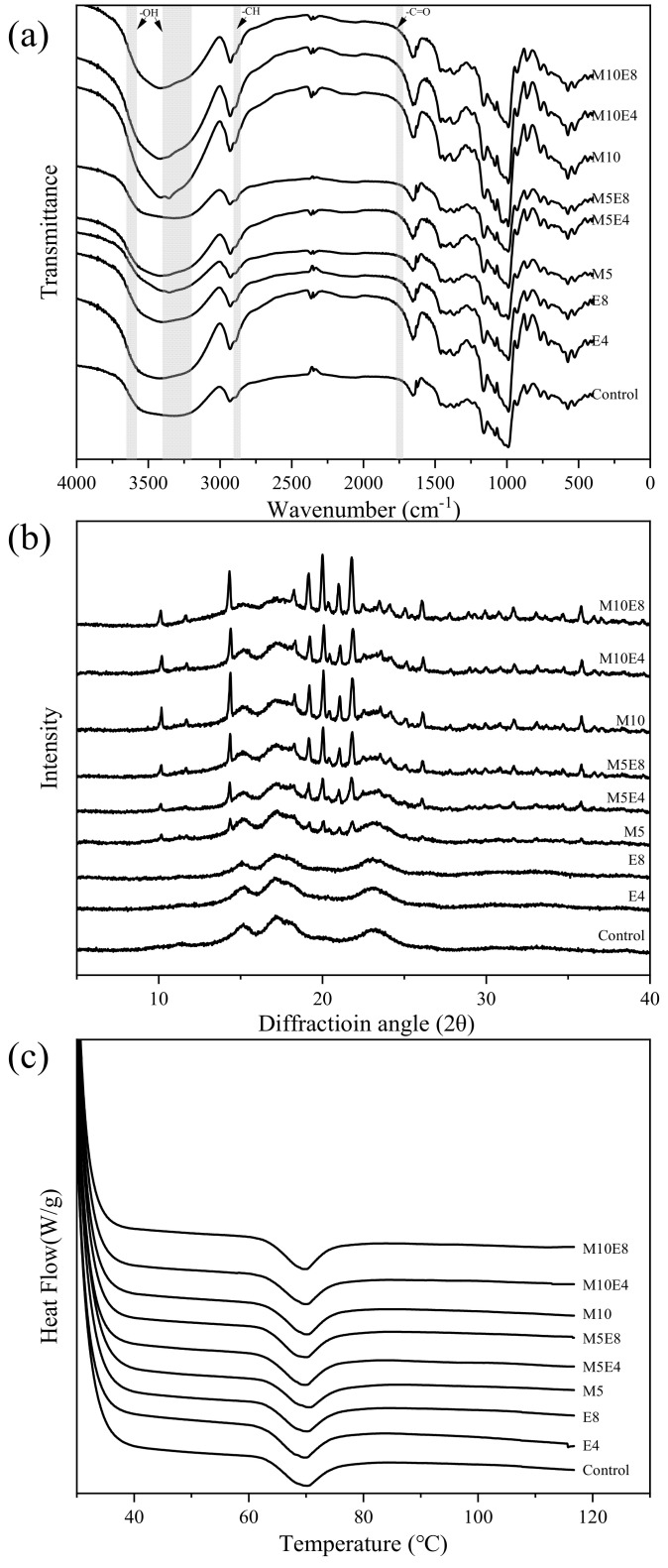
FT−IR (**a**), XRD patterns (**b**), and DSC thermograms (**c**) of lentil starch treated by EBI with different maltose addition proportions.

As shown in Figure 2a, the characteristic morphology of the FTIR curves of lentil starch treated with EBI, with or without maltose, remained unchanged. This indicates that EBI treatment and maltose addition do not introduce new chemical bonds or functional groups, the same as the findings of Yu et al. [24]. The intensity of the telescopic vibrational peaks of –CH (2890 ± 10 cm^−1^) and –C=O (1742 cm^−1^) [25] in starch samples increased after irradiation, related to the change in the glycosidic bond structure. Maltose increased the -OH observed in the peak intensity changes at 3650-3580 cm^−1^ and 3400–3200 cm^−1^ [25]. The peak intensities of –OH in EBI-irradiated lentil starch with maltose were significantly higher than in other zones, suggesting more exposure to –OH after EBI treatment. EBI-irradiated lentil starch had slightly lower –OH peak intensities than maltose-containing samples under the same conditions but were significantly higher than untreated samples (control, M5, and M10). This pattern was confirmed in hydration properties (solubility and swelling power), with samples showing stronger –OH signals having higher solubility and water-holding capacity.

The ratio of the absorption bands at 1047 cm^−1^ and 1022 cm^−1^ (R_1047/1022_) inversely reflects the change in the degree of short-range ordering of starch [26]. Table 1 shows that R_1047/1022_ increased slightly with increasing maltose content for unirradiated starch samples. This may be due to the crystalline nature of maltose enhancing the system’s overall orderliness. After EBI treatment, the R_1047/1022_ values in maltose-added samples were generally lower than in maltose-free samples, indicating that EBI-induced changes in maltose crystals affect the ordered structure of starch crystals, increasing disorder. Luo et al. [16] also reported decreased starch ordering due to the dissociation of non-starch crystalline components during irradiation. Irradiation treatment affects starch by breaking amylose glycosidic bonds and depolymerizing the double-helix structure [16], leading to starch crystal deconstruction and decreasing short-range ordering. However, within certain irradiation limits, the short chains formed by degradation undergo intermolecular rearrangement, forming a more stable structure and enhancing the short-range order of starch [27]. This effect is most pronounced at an irradiation dose of 4 kGy.

Comparing R_1047/1022_ values of M5E4, M10E4, M5E8, and M10E8 shows that irradiated starch samples have higher short-range order with more maltose. EBI does not cause completely amorphized maltose; instead, the energy from maltose crystal dissociation promotes starch short-chain polymerization or rearrangement, an effect that increases with maltose content. Thus, samples with higher maltose content showed greater R_1047/1022_ values.

#### 3.2.2. XRD

As shown in Figure 2b, diffraction peaks characteristic of C-type amylose crystals were observed in native lentil starch at diffraction angles (2θ) of 15°, 17°, 21°, and 23° [28]. The XRD characteristic curves for samples E4 and E8, treated with 4 kGy and 8 kGy EBI, respectively, still exhibited diffraction peaks at these angles. However, the intensity of the peaks was reduced compared to native starch. Samples M5 and M10, which had added maltose but were not treated with EBI, displayed strong diffraction peaks at 2θ angles of 10.2°, 10.9°, 12.7°, 14.4°, 14.8°, 20.1°, 20.4°, and 21.9°. These peaks correspond to anhydrous α-maltose (12.7°, 20.4°, and 21.9°), β-maltose monohydrate (10.2°, 14.4°, and 20.1°), and anhydrous β-maltose (10.9° and 14.8°) [29].

After EBI treatment of starch-containing maltose at 4–8 kGy, the characteristic diffraction peaks of starch at 15°, 17°, 21°, and 23° did not disappear entirely and retained their original shapes, although their intensities were slightly reduced. This indicates that the presence of maltose during EBI treatment enhances starch degradation to a certain extent, increasing the internal disorder of the starch. However, it does not cause substantial amorphization and the crystalline structure of starch is retained. This is further evidenced by the growth ring structure observed in the Maltese cross and CLSM of polarized light microscope images. Moreover, the weakening of the intensity of the starch characteristic peaks became more pronounced with increasing irradiation dose. This suggests that the degree of internal disorder in the starch structure is correlated with the EBI dose.

### 3.3. Chroma Value

Compared to native lentil starch (L* = 97.43, a* = 0.02, and b* = 2.55), starch samples with maltose, without EBI treatment, showed no significant changes in the a* and b* values. However, the brightness (L*) was notably lower with 10% maltose. EBI treatment further reduced the brightness of the samples, causing them to appear more reddish and yellowish, particularly in the presence of maltose. This effect was more pronounced with higher maltose content. The EBI dose and maltose content significantly influenced the chromaticity of the samples. Higher doses of EBI and more significant proportions of maltose resulted in lower L* values and higher b* values, leading to an overall darker yellow color. The chromaticity difference (ΔE) was most significant in the M10E8 sample, with the highest ΔE value of 0.98 compared to native starch.

The observed color changes can be attributed to the caramelization induced by higher EBI doses and the subsequent breakdown of starch molecules. This study found more considerable color differences between samples than previously reported, likely due to the combined caramelization effects of both starch [9] and maltose under irradiation.

### 3.4. Pasting Properties

The pasting characteristics of the samples, including peak viscosity (PV), trough viscosity (TV), final viscosity (FV), breakdown value (BD), setback value (SB), time to peak (PT), and pasting temperature (GT), were analyzed using a rapid viscosity analyzer, with detailed parameters presented in Table 2.

Native lentil starch exhibited high viscosity characteristics across all samples. EBI treatment at 4–8 kGy significantly reduced the PV, TV, BD, FV, and SB values of lentil starch, with higher doses resulting in greater reductions. Interestingly, a significant decrease in PV, TV, BD, FV, and SB was also observed in the presence of maltose, even without EBI treatment. This suggests that maltose alone can reduce the aging tendency of starch. However, when maltose is present, the gel-forming ability, water-holding capacity after gel formation, and shear resistance of starch are further reduced. EBI treatment caused a more pronounced decrease in viscosity in starch-containing maltose.

The reduction in starch paste viscosity after EBI treatment is a typical feature of most irradiation starches [9,13,30]. EBI dissociates water in the sample, generating free radicals that react with glycosidic bonds, leading to the degradation of amylose into shorter linear fragments and the debranching of amylopectin [15]. This increases the lower-molecular-weight fractions of starch, resulting in decreased paste viscosity [13]. The degradation of starch fractions limits the association between starch chains, further reducing paste viscosity [9]. Additionally, irradiation causes the dissociation of some components, such as NaCl [16]. It is hypothesized that a similar dissociation process occurs during EBI treatment of maltose, where the substantial energy released exacerbates the degradation of amylose and amylopectin, ultimately leading to lower viscosity.

### 3.5. Thermal Properties

As shown in Figure 2c, all samples exhibited a single absorption peak of 20–120 °C. The EBI treatment and maltose addition shifted the To, Tp, and Tc to the right compared to native starch. This shift indicates an increase in the thermal stability of the starch samples. Comparing the changes in enthalpy of pasting (ΔH), the presence of maltose and EBI treatment resulted in an increased enthalpy change during starch pasting compared to native lentil starch. Specifically, as the proportion of maltose increased, the ΔH of lentil starch rose from 10.28 J for native starch to 11.7 J for M10. The maximum ΔH value was 13.24 J for M5 at an EBI dose of 4 kGy. Although the ΔH for M10 was slightly lower than that for M5 and E4, it was still higher than that for native lentil starch. When the irradiation dose increased to 8 kGy, the ΔH remained higher than native starch but decreased compared to the 4 kGy treatment.

EBI treatment directly breaks amylose glycosidic bonds, forming short-chained starch fragments. Concurrently, maltose crystals may dissociate during EBI treatment, with the energy released promoting further breaking of glycosidic bonds in starch. The resulting short-chain starch fragments rearrange to form a new stable crystalline structure, making the microcrystals more resistant to melting [23] and enhancing the thermal stability of the modified starch. This increase in ordered structure is also supported by FT-IR results. Consistent with the findings of [9], the increase in ΔH is associated with a decrease in setback in the thermal properties, indicating that EBI treatment reduces the tendency for starch to retrograde in systems containing maltose

### 3.6. Solubility and Swelling Power

The solubility of native lentil starch in water is low, particularly below 70 °C, as shown in Table 3, with values of 0.81% at 50 °C and 1.02% at 60 °C. Upon gradual heating, the solubility at 90 °C significantly increases by 9.94%, from 12.85% to 22.79%, compared to that at 70 °C. Concurrently, the swelling power increases from 7.95 (g/g) to 18.05 (g/g).

The solubility of lentil starch progressively increases with higher doses of EBI. This increase is attributed to EBI’s action on both amorphous and crystalline regions of starch, which breaks glycosidic bonds and degrades the molecular structure, thereby increasing the starch’s contact area with water. Consequently, smaller amylose fragments are more easily leached out, enhancing the solubility of the starch granules [13,16]. Adding maltose further enhances the solubility of both EBI-irradiated and nonirradiated starches. While the increase in solubility is notable, the swelling power also varies slightly with the proportion of maltose added. When treated with the same EBI dose, higher maltose content generally results in higher swelling power. Maltose, being more hydrophilic than starch, increases the soluble matter in the system, thus numerically increasing solubility.

During irradiated treatment, maltose may structurally degrade due to thermal effects and the action of free radicals, leading to cross-linking with starch. The short-chained starch fragments formed by degradation can more effectively hold water molecules, thereby increasing swelling power. At an EBI treatment dose of 4 kGy, lentil starch with 10% maltose exhibits higher swelling power. However, increasing the dose to 8 kGy results in a decrease in swelling power. This suggests that a lower EBI dose (4 kGy) induces cross-linking between maltose and starch, while higher doses disrupt the formed structure. Several factors affect solubility, including interactions between starch chains in crystalline and amorphous regions, the type of starch, the irradiation source, and the irradiation dose [31,32]. The increased swelling power of lentil starch in the presence of maltose may be linked to changes in the crystalline structure of starch, a hypothesis supported by XRD analysis.

### 3.7. In Vitro Digestibility

The microstructure of starch, particularly its amorphous and crystalline regions, significantly influences its enzyme sensitivity. More ordered starch structures present fewer sites for enzyme action [17]. As shown in Table 4, native lentil starch has a high content of resistant starch (RS) at 71.91%, followed by rapidly digestible starch (RDS), and the lowest content of slowly digestible starch (SDS) at 1.75%. EBI significantly increases the RS content, reaching a maximum of 77.68% at an 8 kGy dose, consistent with findings on potato starch by Liang et al. [9]. The anti-digestive properties of starch samples containing maltose decreased with increasing EBI doses. The most significant reduction in SDS + RS content was observed with the addition of 10% maltose, resulting in a 63.88% decrease compared to native starch. At the same irradiation dose, SDS content increased with higher maltose additions despite a reduction in overall SDS + RS content. This indicates that maltose increases the enzyme sensitivity of EBI-modified starch.

The effect of maltose on lentil starch differs slightly from the findings of Luo et al. [16]. The energy from NaCl dissociation led to the formation of short starch chains and enhanced molecular rearrangement, reducing enzyme–starch binding capacity and improving the starch’s anti-digestive properties [33]. However, in this study, RDS and SDS increased significantly in maltose-containing starch treated with EBI. Notably, the SDS content of starch increased significantly after treatment with 4 kGy of EBI, with a more pronounced effect at higher maltose content. There was an overall decrease in RS in treated samples, with higher EBI doses leading to more significant decreases in RS and SDS+RS at the same maltose content. This suggests that maltose exacerbated EBI’s impact on the amorphous and crystalline zones of lentil starch. While more short chains reorganized to form a more ordered structure during irradiation, increasing the SDS content [17], the energy from maltose crystal dissociation also affected the crystalline zones, disrupting their structure and making the starch more susceptible to enzymatic hydrolysis.

### 3.8. PCA

**Figure 3:**
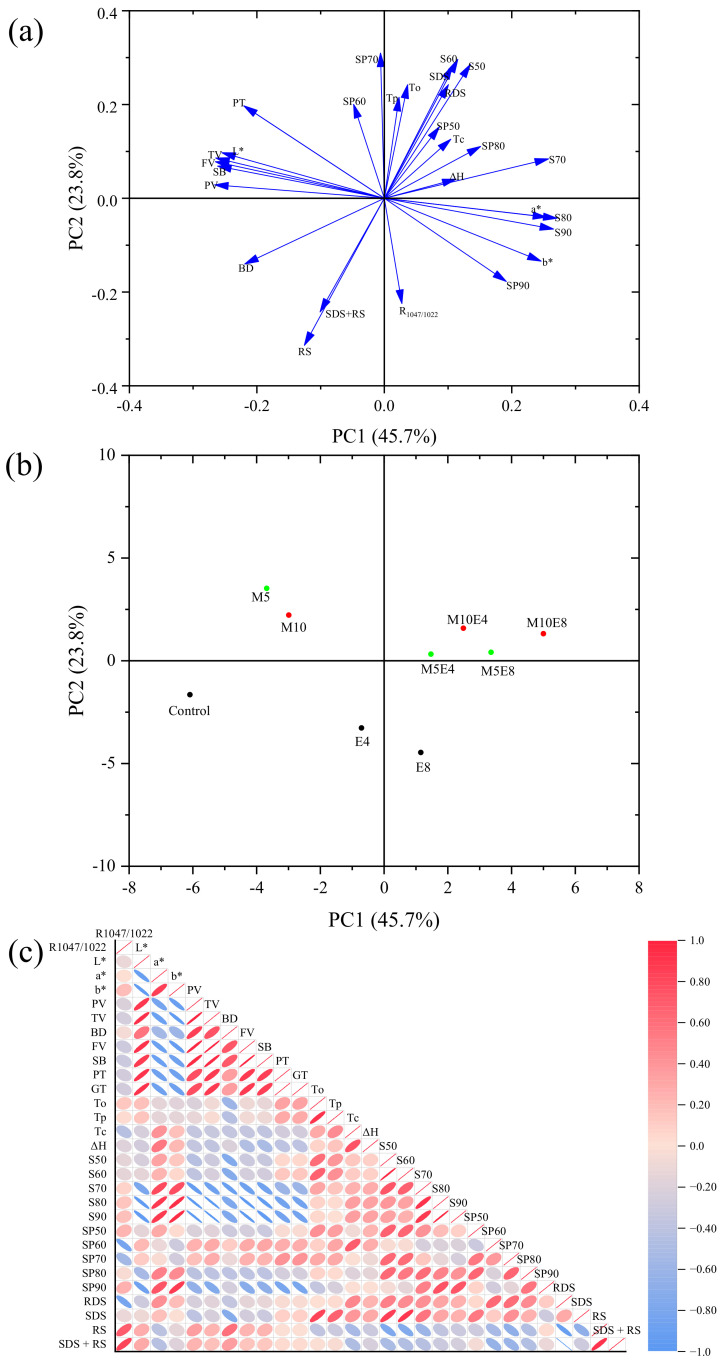
(**a**) score plot, (**b**): loading plot: Principal component analysis (PCA) of physicochemical properties and in vitro digestibility of lentil starch via electron beam irradiation with different maltose addition proportions and correlation analysis of the structure and physicochemical properties of lentil starch by EBI irradiation. (**c**): Pearson’s correlation analysis performed the correlation analysis. The larger the correlation coefficient is, the darker the color will be (red is up-regulated and blue is down-regulated).

The PCA test effectively analyzed and elucidated the potential link between structural changes and physicochemical properties of EBI-irradiated, maltose-containing lentil starch. In the score plot in Figure 3a, samples M5 and M10, containing 5% and 10% maltose without EBI, are in the second quadrant, while native lentil starch is in the third quadrant. Samples containing maltose treated with various doses of EBI shift towards the positive half-axis of the x- and y-axes, concentrating in the first quadrant. Notably, lentil starch treated with 8 kGy EBI has horizontal co-ordinates similar to M10E4, suggesting that 10% maltose content at a 4 kGy EBI dose affects the starch structure similarly to that treated directly at 8 kGy. The co-ordinates of samples with equivalent cumulative absorbed doses were almost identical, indicating they had similar structures and properties. As shown in the loading plot (Figure 3b), the first two principal components (PCs) accounted for 69.5%, 23.8%, and 45.7% of the total variance. Starch solubility and swelling power at 90 °C were positively correlated with R_1047/1022_, suggesting that the degree of short-range ordering significantly influences the starch’s hydration characteristics at higher temperatures.

Although the pasting and digestive properties of lentil starch were in the second and third quadrants, respectively, the acute angle between them indicates that EBI and maltose were positively correlated with the digestive properties of lentil starch. The enthalpy of pasting (ΔH), onset temperature (To), conclusion temperature (Tc), solubility at 50–80 °C, and swelling power at 50 and 80 °C were positively correlated and located in the positive semi-axis of X. This suggests that EBI effectively alters the structure of maltose-containing lentil starch, causing significant changes in its physicochemical properties.

Pearson analysis further elucidates the link between structural changes and property changes in starch. As shown in Figure 3c, R_1047/1022_ positively correlated with RS+SDS and RS content but negatively correlated with SP60 and SP70. To positively correlate with S50, S60, SP50, and SDS, ΔH positively correlated with S70-S90, SP50-60, S50-90, and SDS content. These correlations suggest that changes in the degree of short-range ordering (R_1047/1022_) significantly affect starch digestibility. Specifically, a higher degree of short-range ordering is associated with increased resistant starch (RS) and slowly digestible starch (SDS) content but decreased swelling power at 60 °C and 70 °C (SP60 and SP70).

In addition, starch’s thermal and hydration properties, including solubility and swelling power at 50–90 °C, were negatively correlated with RS content. This indicates that increased thermal stability and hydration properties of starch crystals lead to decreased RS content. In other words, after the EBI of starch-containing maltose, microcrystals are produced, enhancing the solubility and swelling properties of starch in the system. However, the structural changes increase the disorder and enzyme sensitivity of the starch, making it more susceptible to enzymatic decomposition. This results in a decrease in RS content and an increase in digestibility.

## 4. Conclusions

This study systematically investigated the effects of EBI on the structure and physicochemical properties of lentil starch with varying maltose proportions. Higher EBI doses caused significant degradation of starch structure, including growth ring rupture, and reduced fluorescence intensity. Lower EBI doses induced cross-linking and rearrangement of short-chain starch molecules, forming stable structures and improving thermal stability. EBI did not severely damage the surface structure of starch, with or without maltose. Both starch and maltose crystalline properties were retained instead of complete amorphization. However, higher EBI doses further damaged the starch structure by dissociating maltose crystals and producing free radicals. The solubility of EBI-irradiated starch increased with higher maltose content, but the viscosity of starch paste decreased significantly. When maltose exists, it induces cross-linking of short-chain starch during EBI treatment, increasing swelling power. Although resistant starch content decreased with maltose addition, the significant increase in slow-digestible starch content retained the starches’ anti-digestive properties. This study offers new insights for the food industry in producing modified starches with low viscosity, high swelling power, and low digestibility, which are highly suitable for fast food products like instant soups. Additionally, the study demonstrates that EBI treatment of starch-based food products containing maltose can increase the amount of slow-digestible starch in these products.

## Figures and Tables

**Table 1:** EBI treated the FT−IR and chroma values of lentil starch in different maltose addition proportions.

Samples		FT-IR	Chroma Value			
Maltose (%)	EBI (kGy)	1047/1022 cm^−1^	L*	a*	b*	ΔE
0	0	1.025 ± 0.000 ^f^	97.43 ± 0.00 ^c^	0.02 ± 0.01 ^d^	2.55 ± 0.00 ^g^	-
	4	1.063 ± 0.001 ^b^	97.29 ± 0.01 ^d^	0.12 ± 0.01 ^bc^	3.03 ± 0.01 ^e^	0.50 ± 0.02 ^e^
	8	1.085 ± 0.002 ^a^	97.14 ± 0.01 ^f^	0.09 ± 0.01 ^c^	3.30 ± 0.01 ^c^	0.81 ± 0.01 ^c^
5	0	1.018 ± 0.002 ^g^	97.48 ± 0.00 ^b^	0.03 ± 0.01 ^d^	2.50 ± 0.03 ^h^	0.06 ± 0.01 ^g^
	4	1.029 ± 0.002 ^e^	97.17 ± 0.02 ^e^	0.15 ± 0.01 ^a^	2.90 ± 0.03 ^f^	0.46 ± 0.01 ^e^
	8	1.011 ± 0.000 ^h^	97.11 ± 0.01 ^g^	0.15 ± 0.01 ^a^	3.37 ± 0.02 ^b^	0.89 ± 0.01 ^b^
10	0	1.054 ± 0.002 ^c^	97.52 ± 0.00 ^a^	0.03 ± 0.01 ^d^	2.40 ± 0.01 ^i^	0.19 ± 0.01 ^f^
	4	1.053 ± 0.002 ^c^	97.16 ± 0.01 ^ef^	0.13 ± 0.03 ^ab^	3.11 ± 0.04 ^d^	0.61 ± 0.02 ^d^
	8	1.037 ± 0.003 ^d^	97.03 ± 0.01 ^h^	0.16 ± 0.01 ^a^	3.44 ± 0.01 ^a^	0.98 ± 0.03 ^a^

Values are means ± SD. Values with the same letters within the same column are not significantly different (*p* ≤ 0.05).

**Table 2:** Pasting parameters and thermal properties of lentil starch treated by EBI with different maltose addition proportions.

Samples		Pasting Properties							Thermal Properties			
Maltose (%)	EBI (kGy)	PV (cp)	TV (cp)	BD (cp)	FV (cp)	SB (cp)	PT (min)	GT (°C)	T_o_ (°C)	T_p_ (°C)	T_c_ (°C)	ΔH (J/g)
0	0	3182 ± 49 ^a^	1984 ± 57 ^a^	1198 ± 8 ^a^	3545 ± 57 ^a^	1561 ± 0 ^a^	4.30 ± 0.04 ^ab^	4.30 ± 0.04 ^ab^	62.40 ± 0.01 ^i^	69.00 ± 0.00 ^f^	76.74 ± 0.07 ^g^	10.28 ± 0.00 ^i^
	4	2171 ± 1 ^d^	1149 ± 4 ^d^	1022 ± 6 ^b^	1610 ± 7 ^d^	461 ± 11 ^d^	4.17 ± 0.05 ^c^	4.17 ± 0.05 ^c^	62.76 ± 0.01 ^h^	69.29 ± 0.03 ^e^	78.93 ± 0.03 ^e^	12.35 ± 0.03 ^c^
	8	1537 ± 20 ^g^	714 ± 2 ^g^	824 ± 18 ^d^	972 ± 1 ^g^	258 ± 3 ^f^	4.10 ± 0.04 ^c^	4.10 ± 0.04 ^c^	63.28 ± 0.01 ^d^	69.83 ± 0.02 ^b^	78.77 ± 0.09 ^e^	11.19 ± 0.01 ^g^
5	0	2689 ± 39 ^b^	1764 ± 8 ^b^	925 ± 31 ^c^	2942 ± 40 ^b^	1178 ± 33 ^b^	4.37 ± 0.05 ^a^	4.37 ± 0.05 ^a^	63.74 ± 0.00 ^b^	70.35 ± 0.00 ^a^	80.66 ± 0.11 ^c^	11.51 ± 0.05 ^f^
	4	2017 ± 129 ^e^	1080 ± 37 ^e^	937 ± 93 ^c^	1477 ± 66 ^e^	397 ± 29 ^e^	4.10 ± 0.04 ^c^	4.10 ± 0.04 ^c^	62.99 ± 0.00 ^g^	69.63 ± 0.03 ^c^	81.47 ± 0.08 ^a^	13.24 ± 0.03 ^a^
	8	1319 ± 18 ^h^	634 ± 1 ^h^	685 ± 20 ^ef^	837 ± 1 ^h^	203 ± 3 ^g^	4.14 ± 0.09 ^c^	4.14 ± 0.09 ^c^	63.10 ± 0.00 ^f^	69.58 ± 0.07 ^cd^	81.09 ± 0.03 ^b^	13.10 ± 0.01 ^b^
10	0	2390 ± 64 ^c^	1607 ± 30 ^c^	783 ± 35 ^d^	2541 ± 46 ^c^	934 ± 16 ^c^	4.34 ± 0.09 ^a^	4.34 ± 0.09 ^a^	63.80 ± 0.01 ^a^	69.87 ± 0.00 ^b^	78.79 ± 0.06 ^e^	11.70 ± 0.00 ^e^
	4	1705 ± 23 ^f^	939 ± 13 ^f^	766 ± 10 ^de^	1231 ± 18 ^f^	292 ± 5 ^f^	4.20 ± 0.00 ^bc^	4.20 ± 0.00 ^bc^	63.48 ± 0.01 ^c^	69.86 ± 0.01 ^b^	78.00 ± 0.11 ^f^	11.95 ± 0.06 ^d^
	8	1191 ± 15 ^i^	584 ± 4 ^h^	607 ± 18 ^f^	753 ± 8 ^i^	170 ± 5 ^g^	4.13 ± 0.00 ^c^	4.13 ± 0.00 ^c^	63.24 ± 0.01 ^f^	69.55 ± 0.01 ^d^	79.14 ± 0.11 ^d^	10.87 ± 0.08 ^h^

PV, peak viscosity; TV, trough viscosity; BD, breakdown; FV, final viscosity; SB, setback; PT, peak time; GT, pasting temperature. Values are means ± SD. Values with the same letters within the same column are not significantly different (*p* ≤ 0.05).

**Table 3:** The solubility and swelling power of lentil starch treated by EBI with different maltose addition proportions.

Samples		Solubility (%)					Swelling Power (g/g)				
Maltose (%)	EBI (kGy)	50 °C	60 °C	70 °C	80 °C	90 °C	50 °C	60 °C	70 °C	80 °C	90 °C
0	0	0.81 ± 0.04 ^d^	1.02 ± 0.01 ^d^	12.85 ± 0.23 ^f^	20.23 ± 0.37 ^g^	22.79 ± 0.41 ^h^	2.16 ± 0.03 ^b^	2.80 ± 0.06 ^ab^	7.95 ± 0.17 ^abcd^	14.49 ± 0.33 ^b^	18.05 ± 0.43 ^bcd^
	4	1.41 ± 0.16 ^d^	1.12 ± 0.02 ^d^	18.88 ± 0.35 ^d^	28.03 ± 0.52 ^e^	31.90 ± 0.59 ^e^	2.23 ± 0.07 ^ab^	2.75 ± 0.06 ^b^	7.50 ± 0.18 ^de^	14.44 ± 0.39 ^b^	18.77 ± 0.53 ^abc^
	8	1.09 ± 0.07 ^d^	1.23 ± 0.02 ^d^	20.90 ± 0.39 ^c^	33.32 ± 0.62 ^c^	38.30 ± 0.71 ^c^	2.19 ± 0.01 ^ab^	2.73 ± 0.05 ^b^	7.27 ± 0.18 ^e^	13.91 ± 0.40 ^bc^	19.12 ± 0.60 ^ab^
5	0	4.88 ± 0.13 ^c^	5.56 ± 0.10 ^b^	16.39 ± 0.30 ^e^	22.81 ± 0.42 ^f^	25.39 ± 0.46 ^g^	2.22 ± 0.02 ^ab^	2.92 ± 0.06 ^a^	8.25 ± 0.18 ^ab^	14.62 ± 0.35 ^b^	17.81 ± 0.44 ^cd^
	4	5.03 ± 0.37 ^c^	5.26 ± 0.09 ^c^	20.82 ± 0.37 ^c^	31.30 ± 0.57 ^d^	34.28 ± 0.63 ^d^	2.27 ± 0.01 ^ab^	2.86 ± 0.06 ^ab^	7.93 ± 0.19 ^abcd^	14.78 ± 0.40 ^b^	18.49 ± 0.51 ^abc^
	8	5.11 ± 0.63 ^c^	5.85 ± 0.11 ^b^	25.50 ± 0.47 ^b^	35.27 ± 0.64 ^b^	40.47 ± 0.74 ^b^	2.17 ± 0.01 ^ab^	2.85 ± 0.06 ^ab^	7.73 ± 0.19 ^cd^	14.19 ± 0.40 ^b^	18.42 ± 0.56 ^abc^
10	0	8.42 ± 0.24 ^b^	10.34 ± 0.20 ^a^	20.36 ± 0.38 ^c^	24.05 ± 0.45 ^f^	27.12 ± 0.51 ^f^	2.26 ± 0.01 ^ab^	2.76 ± 0.06 ^b^	7.80 ± 0.19 ^bcd^	13.18 ± 0.33 ^c^	16.82 ± 0.44 ^d^
	4	9.68 ± 0.26 ^a^	10.59 ± 0.20 ^a^	25.68 ± 0.48 ^b^	32.07 ± 0.60 ^d^	34.69 ± 0.65 ^d^	2.39 ± 0.25 ^a^	2.73 ± 0.06 ^b^	8.38 ± 0.21 ^a^	18.51 ± 0.50 ^a^	18.79 ± 0.53 ^abc^
	8	10.32 ± 0.11 ^a^	10.60 ± 0.19 ^a^	29.53 ± 0.54 ^a^	38.10 ± 0.69 ^a^	42.25 ± 0.77 ^a^	2.24 ± 0.00 ^ab^	2.79 ± 0.06 ^ab^	8.07 ± 0.21 ^abc^	17.85 ± 0.52 ^a^	19.36 ± 0.60 ^a^

Values are means ± SD. Values with the same letters within the same column are not significantly different (*p* ≤ 0.05).

**Table 4:** The digestive properties of lentil starch treated by EBI with different maltose addition proportions.

Samples		In Vitro Digestion (%)			
Maltose (%)	EBI (kGy)	RDS	SDS	RS	SDS + RS
0	0	26.35 ± 0.79 ^de^	1.75 ± 0.33 ^e^	71.91 ± 0.46 ^b^	73.65 ± 0.79 ^cd^
	4	24.49 ± 0.62 ^ef^	2.17 ± 0.11 ^e^	73.35 ± 0.51 ^b^	75.52 ± 0.62 ^bc^
	8	16.71 ± 1.78 ^g^	5.62 ± 0.62 ^d^	77.68 ± 1.17 ^a^	83.29 ± 1.78 ^a^
5	0	31.62 ± 0.05 ^bc^	8.03 ± 0.27 ^c^	60.36 ± 0.32 ^d^	68.39 ± 0.05 ^ef^
	4	30.58 ± 2.05 ^c^	8.56 ± 0.28 ^c^	60.86 ± 1.76 ^d^	69.42 ± 2.05 ^e^
	8	34.15 ± 0.81 ^ab^	7.99 ± 0.04 ^c^	57.87 ± 0.84 ^e^	65.85 ± 0.81 ^fg^
10	0	23.33 ± 0.66 ^f^	11.82 ± 1.06 ^a^	64.85 ± 1.74 ^c^	76.67 ± 0.66 ^b^
	4	27.53 ± 1.85 ^d^	10.01 ± 0.57 ^b^	62.45 ± 1.29 ^cd^	72.47 ± 1.85 ^d^
	8	36.13 ± 0.32 ^a^	8.95 ± 0.11 ^bc^	54.93 ± 0.21 ^f^	63.88 ± 0.32 ^f^

Values are means ± SD. Values with the same letters within the same column are not significantly different (*p* ≤ 0.05). RS, resistant starch; SDS, slowly digested starch; RDS, rapidly digested starch.

## Data Availability

The original contributions presented in the study are included in the article, further inquiries can be directed to the corresponding author.

## References

[1] Kim H., Baik M. (2021). Pressure moisture treatment and hydro-thermal treatment of starch. Food Sci. Biotechnol..

[2] Aguilera Y., Esteban R.M., Benítez V., Mollá E., Martín-Cabrejas M.A. (2009). Starch, functional properties, and microstructural characteristics in chickpea and lentil as affected by thermal processing. J. Agric. Food Chem..

[3] Di Stefano V., Pagliaro A., Del Nobile M.A., Conte A., Melilli M.G. (2020). Lentil Fortified Spaghetti: Technological properties and nutritional characterization. Foods.

[4] Li D., Fu X., Mu S., Fei T., Zhao Y., Fu J., Lee B., Ma Y., Zhao J., Hou J. (2021). Potato starch modified by *Streptococcus thermophilus* GtfB enzyme has low viscoelastic and slowly digestible properties. Int. J. Biol. Macromol..

[5] Kumar P., Prakash K.S., Jan K., Swer T.L., Jan S., Verma R., Deepika K., Dar M.Z., Verma K., Bashir K. (2017). Effects of gamma irradiation on starch granule structure and physicochemical properties of brown rice starch. J. Cereal Sci..

[6] Wongsagonsup R., Deeyai P., Chaiwat W., Horrungsiwat S., Leejariensuk K., Suphantharika M., Fuongfuchat A., Dangtip S. (2014). Modification of tapioca starch by non-chemical route using jet atmospheric argon plasma. Carbohydr. Polym..

[7] Dar M.Z., Deepika K., Jan K., Swer T.L., Kumar P., Verma R., Verma K., Prakash K.S., Jan S., Bashir K. (2018). Modification of structure and physicochemical properties of buckwheat and oat starch by γ-irradiation. Int. J. Biol. Macromol..

[8] Chen B., Wen Q., Zeng X., Abdul R., Roobab U., Xu F. (2021). Pulsed electric field assisted modification of octenyl succinylated potato starch and its influence on pasting properties. Carbohydr. Polym..

[9] Liang W., Zhao W., Liu X., Zheng J., Sun Z., Ge X., Shen H., Ospankulova G., Muratkhan M., Li W. (2022). Understanding how electron beam irradiation doses and frequencies modify the multiscale structure, physicochemical properties, and in vitro digestibility of potato starch. Food Res. Int..

[10] Zhou X., Ye X., He J., Wang R., Jin Z. (2020). Effects of electron beam irradiation on the properties of waxy maize starch and its films. Int. J. Biol. Macromol..

[11] Vamadevan V., Bertoft E. (2014). Structure-function relationships of starch components. Starch-Stärke.

[12] Rombo G.O., Taylor J.R., Minnaar A. (2004). Irradiation of maize and bean flours: Effects on starch physicochemical properties. J. Sci. Food Agric..

[13] Pan L., Xing J., Zhang H., Luo X., Chen Z. (2020). Electron beam irradiation as a tool for rice grain storage and its effects on the physicochemical properties of rice starch. Int. J. Biol. Macromol..

[14] Jiang F., Du C., Jiang W., Wang L., Du S. (2020). The preparation, formation, fermentability, and applications of resistant starch. Int. J. Biol. Macromol..

[15] Liang W., Zhao W., Liu X., Zheng J., Sun Z., Ge X., Shen H., Ospankulova G., Muratkhan M., Li W. (2023). Investigating the role and mechanism of water in E-beam modified sweet potato starch: Multiscale structure, physicochemical properties, and in vitro digestibility. Food Hydrocoll..

[16] Luo H., Liang D., Liu Q., Zheng Y., Shen H., Li W. (2024). Investigation of the role of sodium chloride on wheat starch multi-structure, physicochemical and digestibility properties during X-ray irradiation. Food Chem..

[17] Ge X., Shen H., Su C., Zhang B., Zhang Q., Jiang H., Yuan L., Yu X., Li W. (2021). Pullulanase modification of granular sweet potato starch: Assistant effect of dielectric barrier discharge plasma on multiscale structure, physicochemical properties. Carbohydr. Polym..

[18] Lei X., Yu J., Hu Y., Bai J., Feng S., Ren Y. (2023). Comparative investigation of the effects of electron beam and X-ray irradiation on potato starch: Structure and functional properties. Int. J. Biol. Macromol..

[19] Díaz O., Ferreiro T., Rodríguez-Otero J., Cobos Á. (2019). Characterization of Chickpea (*Cicer arietinum* L.) Flour Films: Effects of pH and Plasticizer Concentration. Int. J. Mol. Sci..

[20] Englyst H., Kingman S., Cummings J. (1992). Classification and measurement of nutritionally important starch fractions. Eur. J. Clin. Nutr..

[21] Li L., Yuan T.Z., Setia R., Raja R.B., Zhang B., Ai Y. (2019). Characteristics of pea, lentil and faba bean starches isolated from air-classified flours in comparison with commercial starches. Food Chem..

[22] Nakagawa S. (2022). Formation of radicals in irradiated maltose—The role of hydrogen bonding with water. Free. Radic. Res..

[23] Sun X., Sun Z., Guo Y., Zhao J., Zhao J., Ge X., Shen H., Zhang Q., Yan W. (2021). Effect of twin-xuscrew extrusion combined with cold plasma on multiscale structure, physicochemical properties, and digestibility of potato starches. Innov. Food Sci. Emerg. Technol..

[24] Yu Y., Feng M., Wang Q., Liu M., Gao F., Lin S. (2021). Effect of electron beam irradiation on physicochemical properties of corn starch and improvement of enzymatic saccharification of corn starch at high concentration (45%). J. Food Process Eng..

[25] Du Z., Li Y., Luo X., Xing J., Zhang Q., Ren W., Wang L., Chen Z. (2020). Effects of electron beam irradiation on the physicochemical properties of quinoa and starch microstructure. Starch-Stärke.

[26] Chi C., Li X., Lu P., Miao S., Zhang Y., Chen L. (2019). Dry heating and annealing treatment synergistically modulate starch structure and digestibility. Int. J. Biol. Macromol..

[27] Ezekiel R., Rana G., Singh N., Singh S. (2007). Physicochemical, thermal and pasting properties of starch separated from γ-irradiated and stored potatoes. Food Chem..

[28] Majeed T., Wani I.A., Hussain P.R. (2017). Effect of dual modification of sonication and γ-irradiation on physicochemical and functional properties of lentil (*Lens culinaris* L.) starch. Int. J. Biol. Macromol..

[29] Wu Y., Huang W., Cui T., Fan F. (2020). Crystallization and strength analysis of amorphous maltose and maltose/whey protein isolate mixtures. J. Sci. Food Agric..

[30] Xue P., Zhao Y., Wen C., Cheng S., Lin S. (2017). Effects of electron beam irradiation on physicochemical properties of corn flour and improvement of the gelatinization inhibition. Food Chem..

[31] Shen H., Yan M., Liu Y., Liu X., Ge X., Muratkhan M., Ospankulova G., Zhang G., Li W. (2023). Multiscale structure-property relationships of oxidized wheat starch prepared assisted with electron beam irradiation. Int. J. Biol. Macromol..

[32] Shen H., Yu J., Bai J., Liu Y., Ge X., Li W., Zheng J. (2023). A new pre-gelatinized starch preparing by spray drying and electron beam irradiation of oat starch. Food Chem..

[33] Gao S., Liu H., Sun L., Liu N., Wang J., Huang Y., Wang F., Cao J., Fan R., Zhang X. (2019). The effects of dielectric barrier discharge plasma on physicochemical and digestion properties of starch. Int. J. Biol. Macromol..

